# Dual activation of neuronal G protein-gated inwardly rectifying potassium (GIRK) channels by cholesterol and alcohol

**DOI:** 10.1038/s41598-017-04681-x

**Published:** 2017-07-04

**Authors:** Ian W. Glaaser, Paul A. Slesinger

**Affiliations:** Fishberg Dept. of Neuroscience and Friedman Brain Institute, Icahn School of Medicine at Mount Sinai, One Gustave L. Levy Place, New York, NY 10029 USA

## Abstract

Activation of G protein-gated inwardly rectifying potassium (GIRK) channels leads to a hyperpolarization of the neuron’s membrane potential, providing an important component of inhibition in the brain. In addition to the canonical G protein-activation pathway, GIRK channels are activated by small molecules but less is known about the underlying gating mechanisms. One drawback to previous studies has been the inability to control intrinsic and extrinsic factors. Here we used a reconstitution strategy with highly purified mammalian GIRK2 channels incorporated into liposomes and demonstrate that cholesterol or intoxicating concentrations of ethanol, i.e., >20 mM, each activate GIRK2 channels directly, in the absence of G proteins. Notably, both activators require the membrane phospholipid PIP_2_ but appear to interact independently with different regions of the channel. Elucidating the mechanisms underlying G protein-independent pathways of activating GIRK channels provides a unique strategy for developing new types of neuronal excitability modulators.

## Introduction

Many modulatory neurotransmitters in the brain, such as dopamine, acetylcholine, serotonin and GABA, inhibit neuronal activity by stimulating G protein-coupled receptors (GPCRs) that couple to G protein-gated inwardly rectifying (GIRK, also referred to as Kir3) channels^1^. The activation of GIRK channels hyperpolarizes the neuron’s membrane potential, and thereby reduces action potential firing^1, 2^. GIRK channels are widely expressed in the brain, existing as predominantly heterotetramers of three different GIRK channel subunits, GIRK1, GIRK2 and/or GIRK3, or as homotetramers of the GIRK2 subunit^1, 2^. GIRK channels have been implicated in the pathophysiology of several human neurological disorders^3^. Genome-wide association studies (GWAS) of people with schizophrenia have identified single nucleotide polymorphisms (SNPs) in the *Kcnj3* (GIRK1) gene^4^. SNPs in *Kcnj6* (GIRK2) have been linked to alcohol and nicotine dependence, reduced opioid withdrawal and increased opioid requirement for analgesia^5–8^. Recently, a mutation in *Kcnj6* has been proposed to contribute to Keppen-Lubinsky syndrome, a severe developmental disorder with cognitive deficits^9^.

Prior work on GIRK channels has focused on delineating the G protein-dependent pathway for activation of GIRK channels. Following stimulation of GPCRs that couple to pertussis toxin-sensitive G_i/o_ G proteins, the G protein Gβγ subunits bind directly to the channel, and induce a conformational change that opens the channel in a manner that depends on the membrane phospholipid PI(4,5)P_2_ (referred to as PIP_2_)^10–14^. Recently, this interaction was confirmed in an atomic resolution structure based on x-ray crystallography of GIRK2 in complex with Gβγ^15^. Activation of GIRK channels through so-called ‘G protein-independent’ pathways, however, are poorly understood. For example, GIRK channels are activated by alcohol^16–18^ and, similar to Gβγ activation, requires PIP_2_^19^. However, while previous studies with heterologous expression systems and neurons suggested that alcohol directly activates GIRK channels^16–19^, the role of endogenous Gβγ subunits could not be unequivocally dismissed. Furthermore, alternative mechanisms through which alcohol could exert its effects were possible, such as increasing the fluidity of the plasma membrane^20, 21^. Another example of a putative G protein-independent modulator is cholesterol. Cholesterol, which accounts for up to 50% of the total lipid membrane content^22^, has been shown to modulate the activity of potassium channels^23–28^, including cardiac GIRK channels, i.e., GIRK1/GIRK4 subunits^23, 27^. However, whether cholesterol affects brain GIRK channels, comprised mostly of GIRK2-containing tetramers, requires Gβγ subunits, and interacts with alcohol-dependent activation is unknown^1^.

In previous studies using heterologous expression systems, e.g., *Xenopus* oocytes, HEK-293 cells, or neurons, the contribution of endogenous proteins could not be entirely eliminated or controlled^29, 30^. For example, cholesterol concentration in plasma membranes is affected by numerous factors and varies between expression systems^31^. To elucidate more definitively the mechanisms of GIRK activation, a different approach is needed in which the lipid components, G proteins and activators can be all controlled experimentally. In the current study, we implemented a methodology in which we purified GIRK2 channels, reconstituted the channels into liposomes with different lipid compositions and then probed channel function with exogenous ligands. We demonstrate that alcohol and cholesterol directly activate neuronal GIRK2 channels, in the absence of G protein Gβγ subunits but requiring PIP_2_. Interestingly, although PIP_2_ has been considered a co-factor that is necessary, but not sufficient, for channel activation, here we demonstrate that PIP_2_ is sufficient to activate GIRK2 channels in the presence of Na^+^. Further, we discovered that alcohol and cholesterol appear to interact structurally with different regions of the channel. Determining the mechanism of activation of GIRK channels by modulators that bypass G protein-gating could provide a new therapeutic strategy for treating a variety of human diseases.

## Results

### PIP_2_ is necessary and sufficient to activate GIRK2 channels

**Figure 1:**
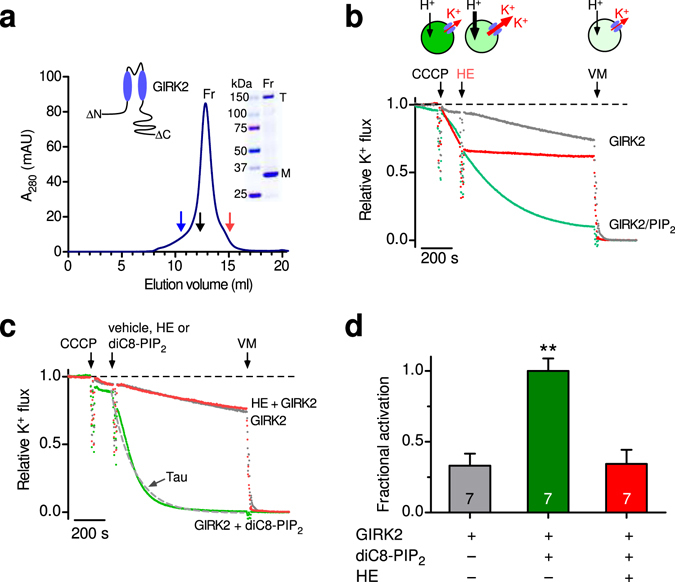
Brain PIP_2_ and soluble PIP_2_ activate reconstituted brain GIRK2 channels in the absence of other proteins. (**a**) Graph shows purification of mouse GIRK2 from *Pichia pastoris*. The absorbance is plotted as a function of elution volume, with molecular weight (MW) standards; Blue - 440 kDa, Black - 158 kDa, Red - 75 kDa (arrows). The major peak elutes at a volume consistent with the size of a GIRK2 tetramer (T). Inset, Coomassie blue staining of a gradient SDS-PAGE protein gel shows two bands, one for tetramer (T) and one for monomer (M). Inset, wild-type GIRK2 was truncated at the amino- and carboxy-termini (see methods). **(b)** Example of K^+^ flux assay with GIRK2 reconstituted into PE/PG liposomes in the absence or presence of brain PIP_2_ (1%) (see methods). The fluorescence is normalized and plotted as a function of time (‘Relative K^+^ flux’). Arrows indicate addition of the proton ionophore carbonyl cyanide m-chlorophenylhydrazone (CCCP), the GIRK2 channel blocker MTS-HE (HE, 100 μM) and valinomycin (VM). Note the quenching of fluorescence for GIRK2 with PIP_2_ (green) upon addition of CCCP, which is attenuated with addition of MTS-HE. Dashed line represents no flux. **(c)** Representative fluorescent traces of GIRK2 alone (grey), GIRK2 and MTS-HE (red), and GIRK2 with acute application of 30 μM diC_8_-PIP_2_ (green). The decay was fit with a single exponential to determine that rate constant (1/τ, s^-1^). **(d)** Bar graph shows the average ( ± SEM) fractional activation with diC_8_-PIP_2_ based on the change in steady-state fluorescence (green bar vs. grey bar). MTS-HE (red bar) significantly inhibits diC_8_-PIP_2_-activated GIRK2 channels (n = 7, P < 0.0001).

Mouse GIRK2 channels were expressed in *Pichia pastoris*, purified, and reconstituted into liposomes of defined compositions (Fig. 1a), following a protocol described previously^15, 32^. GIRK2 channels express efficiently in *Pichia pastoris* and retain their activation by Gβγ subunits^15^. To study K^+^ flux through GIRK2 channels, we used a fluorescence-based K^+^ flux assay^15^ (Fig. 1b) with a plate-reader equipped with a fluidic system to add compounds acutely. In GIRK2-containing liposomes loaded with K^+^ and incubated with the membrane permeable pH-sensitive ACMA dye, the addition of the proton ionophore CCCP results in quenching of the ACMA dye if GIRK channels are open, i.e., H^+^ enter via CCCP if K^+^ can exit the proteoliposome (Fig. 1b). Thus the decrease in fluorescence, i.e., quenching, provides a measure of the relative K^+^ flux that correlates directly with GIRK2 channel activity.

Purified GIRK2 channels, containing amino acids 52 - 380, were reconstituted into liposomes containing 1-palmitoyl-2-oleoyl-sn-glycero-3-phosphoethanolamine (POPE) and 1-palmitoyl-2-oleoyl-sn-glycero-3-phospho-(1’-rac-glycerol) (POPG) at a 3:1 ratio^15^. PE:PG lipids were found previously to support both Gβγ and Na^+^-dependent activation^15^. We first examined the requirement of PIP_2_ for channel activation^12, 14^. PIP_2_ is found in the plasma membrane at concentrations up to 1%^33^ and has been shown previously to be required for GIRK activity^12, 14, 34^. We measured the relative K^+^ flux through GIRK2 channels reconstituted in either the absence or presence of 1% brain PIP_2_, under conditions that saturate the cytoplasmic Na^+^ binding site in GIRK2^35, 36^. GIRK2-containing liposomes containing brain PIP_2_ exhibited a robust quenching of fluorescence upon addition of CCCP (‘GIRK2 /PIP_2_’, Fig. 1b). Subsequent addition of a small molecular inhibitor of GIRK2 channels, MTS-HE (100 μM)^37^, abruptly slowed the rate of quenching, indicating closure of GIRK2 channels (‘GIRK2/PIP_2_ + HE’, Fig. 1b). Similarly, pre-incubating GIRK2-containing liposomes with the K channel inhibitor BaCl_2_ (2 mM) prevented the CCCP-dependent quenching (data not shown). In the absence of PIP_2_, however, there was no significant change in fluorescence, i.e., no quenching, upon CCCP addition (‘GIRK2’, Fig. 1b). Thus, under these defined conditions of PE/PG lipids and cytoplasmic Na^+^, brain PIP_2_ is necessary and sufficient for robust activation of GIRK2 channels^12, 14, 35, 36^.

**Figure 2:**
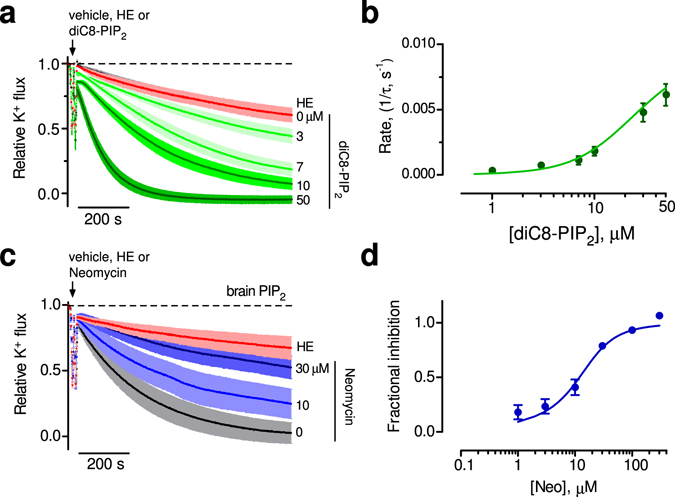
Potency of PIP_2_ activation of GIRK2 channels. (**a**) Normalized fluorescent traces (mean ± SEM) show K^+^ flux for GIRK2 following addition of the indicated concentration of diC_8_-PIP_2_ or MTS-HE (100 μM) (n = 9). (**b**) Plot of the rate of K^+^ flux as a function of diC_8_-PIP_2_ concentration. Line shows best fit using the Hill equation with apparent EC_50_ of 25.1 ± 3.3 μM and Hill coefficient of 1.7 ± 0.1 (n = 10). (**c**) Normalized fluorescent traces (mean ± SEM) show K^+^ flux for GIRK2-containing liposomes with 1% brain PIP_2_ upon addition of the indicated concentration of neomycin, a competitive inhibitor of PIP_2_ (n = 4). (**d**) Plot of the fractional inhibition of K^+^ flux as a function of neomycin concentration. Fraction inhibition was calculated using the apparent steady-state K^+^ flux measurement. Line shows best fit using the Hill equation with IC_50_ for neomycin inhibition 11.1 ± 2.4 μM and with a Hill coefficient of 1.3 ± 0.3 (n = 4).

We next examined whether GIRK2 channels could be directly activated by PIP_2_ applied in real-time. Previous studies demonstrated that analogs of PIP_2_ with shorter tails, e.g., diC_8_-PIP_2_, can also activate GIRK channels^38^. With GIRK2-containing liposomes, the acute addition of diC_8_-PIP_2_ (30 μM) revealed potent activation, i.e., increase in the rate of quenching (Fig. 1c). To quantify the change in K^+^ flux, we measured both the steady state fluorescence at the end of 15 minutes and the rate of quenching following the addition of diC_8_-PIP_2_ (see methods for details). Using both analyses, diC_8_-PIP_2_ clearly activates GIRK2 channels (Figs 1d and 2a,b).

To determine the PIP_2_ sensitivity, we examined the effect of applying different concentrations of diC_8_-PIP_2_ to GIRK2-containing liposomes (Fig. 2a). Note the progressively faster rate of quenching with higher diC_8_-PIP_2_ concentrations. Plotting the rate of K^+^ flux (1/tau) as a function of the diC_8_-PIP_2_ concentration reveals a concentration-dependent increase in the rate of quenching. The best-fit with the Hill equation indicates an apparent EC_50_ of 25.1 ± 3.3 μM and a Hill coefficient of 1.7 ± 0.1 (n = 10) (Fig. 2b). The EC_50_ with diC_8_-PIP_2_ is higher than for that for GIRK2 reconstituted in bilayers in the presence of Gβγ (~15 μM)^34^ and lower than that for GIRK1/GIRK4 channels expressed heterologously (~ 45 μM)^39^.

To probe the sensitivity of GIRK2 channel activation by brain PIP_2_, we examined the effect of neomycin, which is a competitive inhibitor of PIP_2_^40^, on the K^+^ flux with GIRK2 channels. Increasing concentrations of neomycin applied acutely to GIRK2-containing liposomes with brain PIP_2_ attenuated the rate of quenching (Fig. 2c). The IC_50_ for neomycin inhibition is 11.1 ± 2.4 μM (n = 4), with a Hill coefficient of 1.3 ± 0.3 (Fig. 2d). Taken together, these experiments demonstrate that both brain PIP_2_ and soluble diC_8_-PIP_2_ activate GIRK2 channels in a dose-dependent manner, in the absence of G proteins or other ligands, indicating that GIRK2 channels are directly gated by PIP_2_ in the presence of Na^+^.

### Direct action of alcohol on brain GIRK2 channels

**Figure 3:**
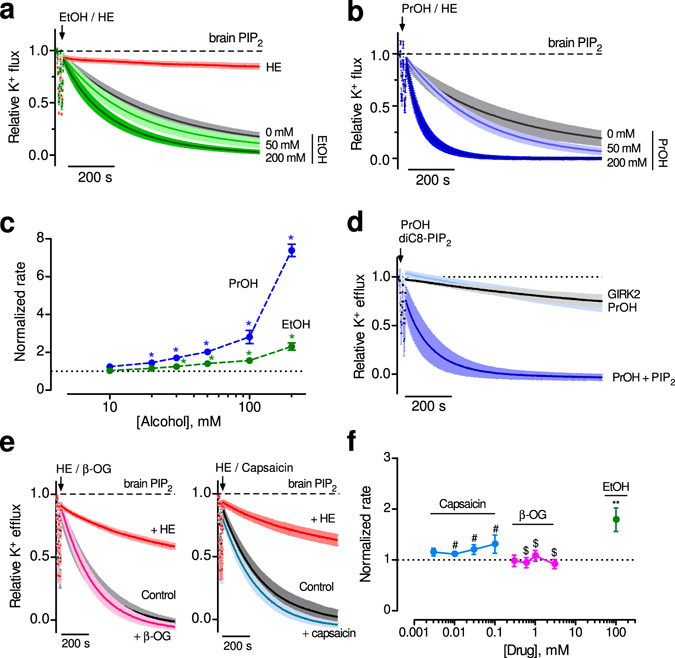
Alcohol directly activates GIRK2 channels in absence of G proteins. (**a,b**) Normalized fluorescent traces (mean ± SEM) show K^+^ flux for GIRK2-containing liposomes with 1% brain PIP_2_ and increasing concentrations of ethanol (EtOH) (**a**) (n = 12) (green traces) or propanol (PrOH) (**b**) (n = 4–6) (blue traces). **(c)** The normalized rate of K^+^ flux is plotted as a function of ethanol (green) and propanol (blue) concentration. Inset, zoom of the dose-response curves over the physiological alcohol concentrations. * p < 0.05 vs. 10 mM using one-way ANOVA and Dunnett’s post hoc test (n = 5–6 for PrOH and 11–12 for EtOH). **(d)** Normalized fluorescent traces (mean ± SEM) show K^+^ flux for GIRK2 in the presence of 100 mM PrOH in the absence (light blue, n = 3) or presence of 50 μM diC8-PIP_2_ (dark blue, n = 4). GIRK2 in the absence of both PrOH and diC_8_-PIP_2_ is shown for comparison (black, n = 4). (**e)** Normalized fluorescent traces (mean ± SEM) show K^+^ flux for GIRK2-containing liposomes with 1% brain PIP_2_ and 1 mM β-OG (**left**) (n = 4, magenta) or 30 μM capsaicin (**right**) (n = 4, blue). MTS-HE inhibition is shown for comparison (red). **(f)** The normalized rate of K^+^ flux is plotted as a function of different β-OG (pink) or capsaicin (blue) concentrations, and is compared to 100 mM EtOH (green). ^#^not significant vs. 3 μM Capsaicin, ^$^not significant vs. 0.3 mM β-OG, ** P < 0.05 vs. 3 μM Capsaicin or 0.3 mM β-OG using one-way ANOVA and Dunnett’s post hoc test.

In addition to G proteins, GIRK channels are activated by alcohols^16–18^. In order to address whether this property is retained in the purified protein, we examined the effect of directly applying ethanol to GIRK2-containing liposomes with brain PIP_2_. Ethanol, added at increasing concentrations, produced a dose-dependent increase in the relative rate of K^+^ flux (Fig. 3a). Similarly, increasing concentrations of propanol also enhanced the relative rate of K^+^ flux (Fig. 3b). We calculated the change in the rate of K^+^ flux, relative to 0 mM alcohol, and plotted the normalized rate as a function of alcohol concentration (Fig. 3c). Note the increase in the normalized rate of K^+^ flux with concentrations greater than 10 mM, a rank order of activation with propanol greater than ethanol, and no apparent saturation at 200 mM. These properties of alcohol activation are remarkably similar to those with GIRK2 channels expressed heterologously in HEK293 cells^16–18^. Importantly, alcohol-dependent activation required brain PIP_2_ (Fig. 3d). Thus, we show for the first time that alcohol-dependent activation of GIRK channels requires only PIP_2_, and no other proteins, e.g., Gβγ subunits.

Using the lipid-reconstitution system, we could now investigate the possibility that changes in lipid membrane dynamics could affect GIRK2 function^20, 21^. We tested the effect of compounds demonstrated previously to increase membrane elasticity, e.g., β-octyl glucoside (β-OG) and capsaicin^41, 42^. Direct application of β-OG (1 mM) or capsaicin (30 μM) did not alter the rate of K^+^ flux for GIRK2 channels reconstituted into liposomes with brain PIP_2_ (Fig. 3e). There was no significant change in the rate of K^+^ flux with increasing concentrations of β-OG or capsaicin, in contrast to the change in K^+^ flux with ethanol (Fig. 3f). Taken together, these experiments demonstrate that known lipid-membrane disruptors have little effect on GIRK2 channel activity, providing additional support for the direct activation of GIRK2 by ethanol.

### Direct action of cholesterol on brain GIRK2 channels

**Figure 4:**
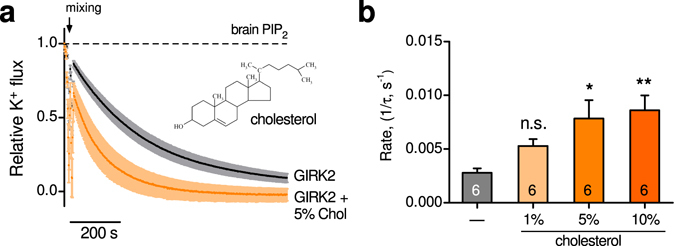
Cholesterol directly activates GIRK2 channels. (**a**) Normalized fluorescent traces (mean ± SEM) show K^+^ flux for GIRK2-containing liposomes with 1% brain PIP_2_ in the absence (black) or presence of 5% cholesterol (orange) (n = 6). Inset, chemical structure of cholesterol is shown. **(b)** Bar graph shows the increase in the rate of K^+^ flux with different cholesterol concentrations (1%, 5%, and 10%). Statistical significance * p < 0.05, ** p < 0.01 (n = 6).

Cholesterol inhibits constitutively open inwardly-rectifying potassium channels^25, 26^ but appears to activate cardiac (GIRK1/GIRK4) and brain (GIRK2) channels^23, 27, 43^. Whether cholesterol acts directly on brain GIRK2 channels in the absence of Gβγ is unknown. Using GIRK2-containing liposomes that have brain PIP_2_ (1%), we examined the effect of cholesterol on the relative K^+^ flux. The basal rate of K^+^ flux through GIRK2 channels was significantly enhanced in membranes containing 5% cholesterol (Fig. 4a). Both 5% and 10% cholesterol significantly increased the relative rate of K^+^ flux, while 1% was ineffective (Fig. 4b **)**. Interestingly, the relative rate of K^+^ flux was similar for 5% and 10% cholesterol, suggesting near saturation for this response.

**Figure 5:**
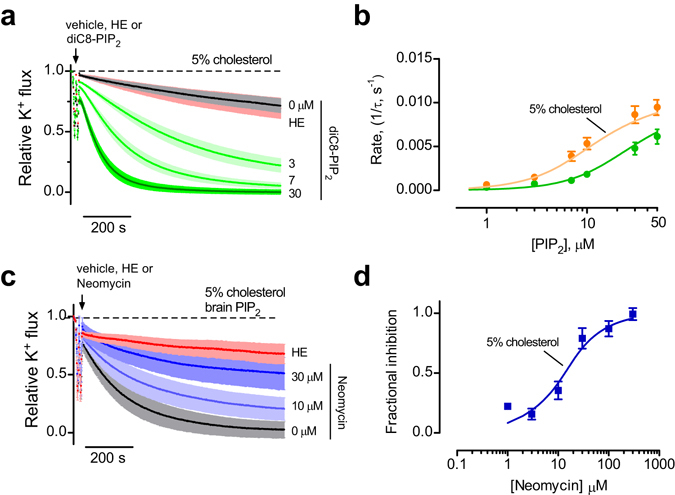
Cholesterol enhances sensitivity of GIRK2 channels to PIP_2_. (**a**) Normalized fluorescent traces (mean ± SEM) show K^+^ flux for GIRK2-containing liposomes with 5% cholesterol in response to increasing concentrations of diC_8_-PIP_2_ (green) or MTS-HE (100 μM, red). Note little change in K^+^ flux in the absence of PIP_2_ (black) similar to that of liposomes without cholesterol (n = 7). **(b)** The rate of K^+^ flux is plotted as a function of different diC_8_-PIP_2_ concentrations for GIRK2 in the presence of 5% cholesterol (mustard circles). Line shows best fit using the Hill equation with EC_50_ of 12.2 ± 2.5 μM and a Hill coefficient of 1.5 ± 0.2 (n = 6). For reference, dose-response without cholesterol from Fig. 2B is shown (green circles). **(c)** Normalized fluorescent traces (mean ± SEM) show K^+^ flux for GIRK2-containing liposomes with 1% brain PIP_2_ / 5% cholesterol with different concentrations of neomycin (n = 5). **(d)** The rate of K^+^ flux for GIRK2/brain PIP_2_/5% cholesterol is plotted as a function of different neomycin concentrations. Line shows best fit using the Hill equation with an IC_50_ 15.0 ± 4.6 μM and Hill coefficient of 1.2 ± 0.3 (n = 5).

GIRK2 channels reconstituted in cholesterol-containing liposomes (5%) lacking brain PIP_2_ exhibited no basal K^+^ flux, indicating that PIP_2_ is required for cholesterol activation, similar to alcohol activation. We hypothesized that cholesterol may enhance GIRK2 activity by altering the sensitivity to PIP_2_. To test this, we measured the rate of K^+^ flux in GIRK2-containing liposomes containing 5% cholesterol that were directly exposed to different concentrations of diC_8_-PIP_2_ (Fig. 5a). The dose-response curve for diC_8_-PIP_2_ in the presence of cholesterol shifted to lower concentrations, compared to that without cholesterol (Fig. 5b). The EC_50_ for PIP_2_ activation of GIRK2 in the presence of cholesterol was 12.2 ± 2.5 μM (n = 6), compared to an EC_50_ of 25.1 ± 3.3 μM without cholesterol. Thus, the apparent affinity for PIP_2_ increases nearly 2-fold in the presence of cholesterol. In GIRK2 proteoliposomes containing both brain PIP_2_ (1%) and 5% cholesterol, acute application of neomycin decreased the rate of K^+^ flux, with an IC_50_ of 15.0 ± 4.6 μM (Fig. 5c,d). The IC_50_ for neomycin inhibition is indistinguishable from that measured in the absence of cholesterol (Fig. 2d), suggesting little difference in the off-rate of PIP_2_. Thus, in addition to alcohol, cholesterol appears to directly activate GIRK2 channels through enhancement of the interaction with PIP_2_.

### Cholesterol does not affect the alcohol sensitivity of GIRK2 channels

**Figure 6:**
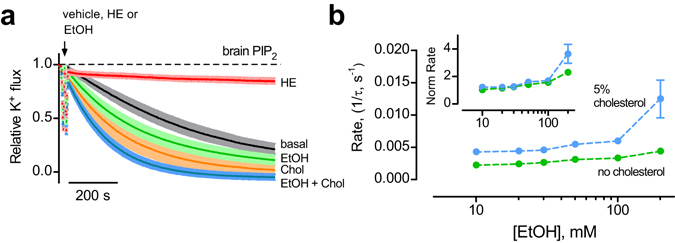
Cholesterol does not affect the ethanol sensitivity of GIRK2 channels. (**a**) Normalized fluorescent traces (mean ± SEM) show the K^+^ flux for GIRK2-containing liposomes with 1% brain PIP_2_ (black trace), and either 100 mM EtOH (green), 5% cholesterol (orange), or both 100 mM EtOH and 5% cholesterol (blue trace) (n = 4–12) (**b**) The rate of K^+^ flux for GIRK2 with PIP_2_ and 5% cholesterol (blue) or PIP_2_ alone (green) is plotted as a function of different EtOH concentrations. Inset, the normalized rate of K^+^ flux is plotted as a function of EtOH concentration.

Both alcohol and cholesterol activate GIRK2 channels by increasing the apparent affinity for PIP_2_. We next investigated the interaction between alcohol and cholesterol on GIRK2 activation. With GIRK2-containing liposomes in 5% cholesterol (a saturating concentration in our experiments), ethanol enhanced the relative K^+^ flux to a greater extent than for cholesterol alone or ethanol alone (Fig. 6a). The rate of K^+^ flux with 5% cholesterol increased over a range of ethanol concentrations (Fig. 6b). However, the dose-response curves completely overlap over the physiological range of activation, e.g., 10–100 mM, after adjusting for the choleseterol-dependent increase in the basal K^+^ flux (Fig. 4b), (Fig. 6b **, inset**). These results indicate that the effects of alcohol and cholesterol on GIRK channel are additive, suggesting these two modulators increase channel activity via separate structural sites in the channel.

## Discussion

GIRK channels provide a major pathway for inhibition in the brain that is important in both normal and diseased states. The canonical pathway for GIRK channel activation is mediated by G protein Gβγ subunits, which occurs following stimulation of a G_i/o_-coupled GPCRs, such as GABA_B_, dopamine D2 or mu opioid receptors^1, 2^. Recent crystal structures of GIRK2 in complex with Gβγ and PIP_2_ have provided structural and mechanistic details of how Gβγ associates with GIRK channels^15, 32^. It has become increasingly evident that G protein-independent pathways also exist for modulating GIRK channels; however, much less is known about the mechanism of action. A majority of the identified compounds that modulate GIRK channels independently of G proteins appear to inhibit GIRK channel activity^44–48^. Conversely, the number of drugs that directly activate GIRK channels is small^16, 17, 49, 50^. Here, we demonstrate for the first time that ethanol and cholesterol directly activate GIRK2 channels via changes in the interaction with PIP_2_, and do not require the presence of G proteins or other endogenous proteins.

In previous studies, extracellular application of alcohol enhanced GIRK channel activity, both with heterologously expressed channels, or with natively expressed channels in cerebellar neurons^16, 17^. Alcohol activation was determined to be insensitive to pertussis toxin (PTX) or antibodies to the G protein β1 subunit, suggesting that G proteins were not involved^17^. Further, deletion studies^16^, the identification of an alcohol pocket in x-ray crystallographic structures^18, 51^ and alcohol-tagging studies^19^ all indicated that alcohol activation was likely mediated by the direct interaction of alcohol with the channel. However, the involvement of endogenous proteins, e.g. Gβγ subunits, could not be ruled out with heterologous expression systems, such as oocytes or HEK293 cells, or native cell systems, i.e., neurons. In addition, a component of ethanol activation could include an alteration in the lipid bilayer fluidity^21^, since this was not directly tested. By reconstituting purified GIRK2 channels in liposomes, controlling the components of the bilayer, and adding alcohol in isolation of any other proteins, we could overcome these previous limitations and demonstrate that intoxicating concentrations of ethanol, i.e., >20 mM, directly activate GIRK2 channels in the presence of PIP_2_. For reference, a blood alcohol level of 0.08% is ~ 17 mM, binge-drinking levels can reach 50 mM, and the anesthetic concentration is around 190 mM^52^. On the other hand, we find that two types of lipid disruptive amphiphiles, e.g., β-octyl glucoside (β-OG) and capsaicin, do not activate GIRK2 channels. Thus, the mechanism of ethanol activation of GIRK2 channels is unlikely to involve an indirect component from an alcohol-dependent changes in the membrane lipids or from Gβγ subunits.

Using mammalian GIRK2 channels reconstituted in defined lipid membranes, we also determined that cholesterol directly activates GIRK2 channels. By contrast, cholesterol inhibits the activity of many different types of ion channels, including other members of the inwardly rectifying potassium channel family (Kir)^24, 26, 53, 54^. Like alcohol, cholesterol activation of GIRK2 depends on the presence of PIP_2_. Both activators appear to increase the apparent affinity of the channel for PIP_2_, similar to Gβγ activation^34^, suggesting that a tighter association of PIP_2_ with the channel may contribute to the increase in channel activity. In addition, we demonstrate that PIP_2_ is not only necessary, but is sufficient to activate GIRK2 channels with cytoplasmic Na^+^ in the absence of Gβγ subunits. As such, PIP_2_ could be considered an ‘agonist’ for GIRK channels. Moreover, molecules typically described as channel activators, i.e., Gβγ subunits and alcohol, could be mechanistically referred to as positive allosteric modulators (PAMs). PAMs are molecules that cannot activate the channel on their own but increase the efficacy or potency of an agonist, i.e., PIP_2_. For example, benzodiazepine is a PAM for GABA_A_ receptors; it does not activate GABA_A_ receptors but shifts the EC_50_ for agonist, i.e., GABA, activation^55^. However, the endogenous levels of PIP_2_ are low relative to the sensitivity of GIRK channels, resulting in a small basal channel activity. GIRK channels are considered to have a relatively low affinity for PIP_2_, compared to constitutively active Kir2.1 channels^56^. Consistent with this, the EC_50_ for activation of GIRK2 with diC_8_-PIP_2_ is ~25 μM, which compares to a 10-fold lower EC_50_ of ~2 μM for Kir2.1^57^. Interestingly, the GIRK channel activators, i.e., Gβγ, alcohol and cholesterol, do not alter the levels of PIP_2_ but instead change the apparent affinity of the channel for PIP_2_. The shift in apparent PIP_2_ affinity with these activators likely occurs through allosteric changes in the channel that alter the rates of association and dissociation of PIP_2_. On the other hand, stimulation of GPCRs that couple to Gq G proteins can reversibly lower the levels of membrane PIP_2_, leading to a decrease in GIRK channel activity^58^.

Similar to its effects on brain GIRK2 channels, cholesterol enhances acetylcholine activated potassium (K_ACh_) channels in the heart, which are composed of GIRK1 and GIRK4 subunits^23, 27^. Interestingly, this effect was proposed to be independent of Gβγ but with little change in the apparent PIP_2_ affinity^23^. In these experiments, the relative association of PIP_2_ with GIRK1/GIRK4 heteromeric or GIRK4* homomeric channels was inferred from measuring the rate of current inhibition following activation of a PIP_2_-depleting phosphatase^23^. The rate was similar in the absence and presence of cholesterol^23^. By contrast, we find that cholesterol activates the neuronal GIRK2 channel activity by increasing the apparent association with PIP_2_ in the absence of Gβγ subunits. Whether this difference in PIP_2_ dependence is unique to GIRK2 remains to be determined.

A high-resolution structural view of the binding site for cholesterol in GIRK2 is not available. By contrast, a structural pocket has been identified in GIRK2 channels for alcohol activation^18, 51^. The alcohol pocket is located at the interface of the cytoplasmic domain of two adjacent channel subunits, and is formed by hydrophobic amino acids in the N-terminal domain, the βL-βM strands, and the βD-βE strands^18^. This binding pocket overlaps with the region involved in Gβγ subunits activation^15, 59^. Molecular dynamics (MD) simulations combined with functional studies have suggested cholesterol may be coordinated by residues in the transmembrane spanning regions^60, 61^. Recently, Bukiya *et al*. (2017) conducted cholesterol-docking experiments and identified four amino acids that potentially interact directly with cholesterol, V99, V101, L174 and L183. Interestingly, these sites are physically close to the binding pocket for PIP_2_ in the GIRK2 closed structure^32^, raising the possibility that cholesterol may enhance the access of PIP_2_ to the channel. Thus, the site for cholesterol modulation is unlikely to be the same as that for alcohol (i.e. membrane vs. cytoplasmic). In addition, we noted functional differences between alcohol and cholesterol activation. First, there was no apparent shift in ethanol sensitivity in the presence of cholesterol. A direct or allosteric effect of alcohol on cholesterol activation might be reflected in differences in the dose-response curves. Second, the basal rate of K^+^ flux through GIRK2 channels in the presence of alcohol and a saturating level of cholesterol was higher than the rate for alcohol alone. Together, these results suggest that alcohol and cholesterol interact structurally and mechanistically with different regions of the channel.

Cholesterol is ubiquitous in plasma membranes, accounting for up to 50% of the lipid mass^22^. Changes in brain cholesterol are implicated in numerous neurodegenerative diseases, such as Alzheimer’s, Huntington’s, Parkinson’s, and Niemann-Pick disease^62^. Cholesterol metabolism and utilization in the brain is significantly different than the rest of the body. Although the brain only makes up 2-5% of the body mass, almost 25% of the total body cholesterol resides in the brain^63^. Moreover, cholesterol degradation in the brain is low. An increase of cholesterol in the brain has been reported to decrease the firing rate in hippocampal neurons, suggesting it may reduce excitability^64^. Notably, some types of GIRK channels are localized in lipid rafts, which are enriched in cholesterol^65, 66^. Thus, changes in membrane cholesterol content may alter the basal activity of neuronal GIRK channels. Consistent with this, Bukiya *et al*. (2017) reported that cholesterol enrichment increased baclofen-induced, tertiapin-inhibited GIRK-like currents in hippocampal neurons. Similarly, cholesterol (33%) enhanced the basal activity of a constitutively-active mutant GIRK2 channel^43^.

GIRK channels are emerging as a potential drug target for treating alcoholism and other neurological disorders^3^. Interestingly, chronic alcohol exposure leads to increased cholesterol levels and more widespread distribution of cholesterol in the membrane leaflets^67, 68^. Moreover, increases in cholesterol have been also reported in cases of fetal alcohol exposure^69^. The potentiation of GIRK channel activity by cholesterol could amplify the effects of alcohol, perhaps contributing to a comorbidity of cholesterol and alcohol. With a better understanding of the molecular mechanism underlying the direct activation of GIRK channels by small compounds like ethanol and cholesterol, it should be possible to design new drugs that specifically modulate the activity of GIRK channels through these G protein-independent pathways.

## Methods

### Molecular Biology

We used a variant of GIRK2 shown previously to express efficiently in *Pichia pastoris*; mouse GIRK2 (containing amino acids 52–380) is linked in-frame with an HRV 3 C protease site, green fluorescent protein (GFP) and a decahistidine (HIS10) tag (a generous gift from R. MacKinnon, The Rockefeller University, New York, NY). GIRK2 clones in pPICZ were transformed into *Pichia pastoris* using the lithium chloride transformation method^70^ or electroporation (according to manufacturer protocols). Transformants were screened based upon Zeocin resistance (>500 μg/ml) and GFP emission. Highest expressing clones were selected for large-scale purification.

### Protein purification and reconstitution

All GIRK channels were expressed and purified in *P. pastoris* as described previously^15, 32^. Briefly, the highest-expressing clone was grown in BMGY medium and induced in BMM medium containing 1% methanol. Cells were harvested, resuspended in buffer (50 mM HEPES, pH 7.4; 150 mM KCl; 1 mM TCEP; 1 mM AEBSF and Complete EDTA-free protease inhibitor tablets (Roche)), dripped into liquid nitrogen, and placed at −80 °C. Frozen cells were lysed in a Mixer Mill (Retsch) 5-times for 3 minutes at 25 Hz and solubilized in 50 mM HEPES, pH 7.35; 150 mM KCl; 1 mM TCEP; 1 mM AEBSF; 3% (w/v) n-Dodecyl-β-D-maltoside (DDM; Anatrace) and Complete ULTRA EDTA-free protease inhibitor tablets (Roche) with gentle stirring at 4 °C. Unsolubilized material was separated by centrifugation at 40,000 × g for 40 min at 4 °C. The supernatant was injected onto a HISTrap HP column (GE Healthcare) equilibrated in wash buffer (50 mM HEPES, pH 7.0; 150 mM KCl; 0.4% DDM; 20 mM imidazole) connected to an ÄKTA pure (GE Healthcare) chromatography system and eluted in buffer containing 300 mM imidazole. The HISTrap column eluate was pooled, exchanged into imidazole-free buffer and digested overnight at 4 °C with HRV 3 C protease, purified as described^71^ (a generous gift of Daniel Minor, UCSF, San Francisco, CA). Digested protein was concentrated and run on a Superdex-200 gel filtration column in 20 mM TRIS-HCl pH 7.5, 150 mM KCl, 0.1% (w/v) DDM (anagrade), 5 mM DTT, and 1 mM EDTA. Fractions eluting at a volume consistent with the GIRK channel tetramer were pooled, concentrated and examined by SDS-PAGE and Coomassie blue staining. We also analyzed the purified protein using tandem mass spectrometry and detected significant peptides for only GIRK2 and the mass spectrometry experimental artifacts trypsin and keratin. No other proteins were evident.

Purified GIRK2 channels were reconstituted into lipid vesicles as described previously^15, 34^. Briefly, a lipid mixture containing 1-palmitoyl-2-oleoyl-*sn*-glycero-3-phosphoethanolamine (POPE), 1-palmitoyl-2-oleoyl-*sn*-glycero-3-phospho-(1’-*rac*-glycerol) (POPG), and L-α-phosphatidylinositol-4,5-bisphosphate (Brain, PI(4,5)P_2_ (Porcine)) at mass ratios of 3:1:0.04 (POPE:POPG:PIP_2_) or 3:1 (POPE:POPG) was prepared, reconstituted in vesicle buffer (20 mM K-HEPES, pH 7.4; 150 mM KCl; 0.5 mM EDTA containing 35 mM CHAPS) and incubated with protein in detergent at a 1:200 protein: lipid ratio unless otherwise indicated. Where indicated, cholesterol (Ovine wool) was added to vesicles at a mole percentage of 1%, 5% or 10%. Cholesterol could not be incorporated into liposomes at concentrations higher than 10% as noted previously^25^. Detergent was removed through sequential addition of Bio-beads SM-2 (Bio-rad). All phospholipids, cholesterol, and Brain PIP_2_ were purchased from Avanti Polar Lipids, Inc. Soluble PIP_2_ (diC_8_-PIP_2_) was purchased from Echelon Biosciences.

### Flux assay

Liposomes were diluted 1:20 into flux buffer (20 mM Na-HEPES, pH 7.4; 150 mM NaCl; 0.5 mM EDTA) containing 5 μM of the H^+^ sensitive dye 9-Amino-6-chloro-2-methoxyacridine (ACMA). Fluorescence was measured using a Flexstation 3 microplate reader (Molecular Devices) with the following parameters: 410 nm excitation, 480 nm emission, 455 nm cutoff, medium PMT sensitivity, and sampling at 2 seconds. After a stable baseline fluorescence (150 s) was obtained, the H^+^ ionophore *m*-chlorophenyl hydrazone (CCCP) was automatically added (1 μM), then a second addition consisting of different compounds or vehicle was added 150 s later, followed 900 s later by a third addition with the K^+^ ionophore Valinomycin (100 nM), to determine the maximal K^+^ flux. GIRK2 channels are likely arranged in both orientations in the liposomes. However, we expect the channels oriented inside-out to support high K^+^ flux because of high Na^+^ in the flux buffer and high K^+^ in the liposome^14, 35, 36^.

### Data Analysis

To compare between different flux assay runs, the fluorescence was either normalized to the baseline fluorescence before CCCP (*F*_*b*_) and the fluorescence following Valinomycin (*F*_*v*_), or in the case of acutely adding a compound, the fluorescence after CCCP (*F*_*b*_) and the fluorescence following Valinomycin (F_v_). This normalized fluorescence (*F*_*N*_), defined as ‘relative K^+^ flux”, was calculated according to the equation (Eq. 1):

1 $${F}_{N}(t)=\frac{F(t)-{F}_{v}}{{F}_{b}-{F}_{v}}$$

where *F*_*N*_ *(t)* is the normalized fluorescence at time t and *F(t)* is the relative fluorescence unit (RFU) as a function of time.

The rate of K^+^ flux (1/τ) was determined by fitting the normalized fluorescence decay with a single exponential (Eq. 2):

2 $${F}_{N}(t)={F}_{0}{e}^{\frac{-t}{\tau }}+C$$

where F_o_ is the amplitude, 1/τ is the rate, and C is a constant. In experiments where it was not possible to fit the decay, the amplitude of the fluorescence just prior to adding valinomycin (1190 s) was used to calculate the fractional inhibition of K^+^ flux.

Dose-response curves for diC_8_-PIP_2_/neomycin were fit to the Hill equation (Eq. 3):

3 $$y=\frac{{y}_{max}}{1+{(\frac{{x}_{50}}{[x]})}^{h}}$$

where y_max_ is the maximal flux rate or fractional inhibition, [X] is the concentration, h is the Hill coefficient, and *x*_50_ is the half-maximal effective concentration, i.e. EC_50_ for PIP_2_ or IC_50_ neomycin. Dose response curves for modulators, e.g., alcohol, β-OG, capsaicin, do not reach saturation and were normalized to the basal flux rate in the absence of ligand. All values are reported as mean ± S.E.M, with statistical significance (*P* < 0.05) determined using one-way ANOVA followed by Dunnett’s multiple comparison post hoc test unless otherwise noted. All data were analyzed using Excel (Microsoft) and Prism (GraphPad).
